# Human Monoclonal IgE Antibodies—a Major Milestone in Allergy

**DOI:** 10.1007/s11882-022-01055-w

**Published:** 2022-12-02

**Authors:** Scott A. Smith, Maksymilian Chruszcz, Martin D. Chapman, Anna Pomés

**Affiliations:** 1 Department of Medicine, Vanderbilt University Medical Center, Nashville, TN 37232 USA; 2 Department of Chemistry and Biochemistry, University of South Carolina, Columbia, SC 29208 USA; 3 InBio, Charlottesville, VA 22903 USA

**Keywords:** Human IgE monoclonal antibody, Allergy, Allergens, Conformational epitope, X-ray crystallography, Diagnosis

## Abstract

**Purpose of Review:**

Bound to its high affinity receptor on mast cells and basophils, the IgE antibody molecule plays an integral role in the allergic reaction. Through interactions with the allergen, it provides the sensitivity and specificity parameters for cell activation and mediator release that produce allergic symptoms. Advancements in human hybridoma technologies allow for the generation and molecular definition of naturally occurring allergen-specific human IgE monoclonal antibodies.

**Recent Findings:**

A high-resolution structure of dust mite allergen Der p 2 in complex with Fab of the human IgE mAb 2F10 was recently determined using X-ray crystallography. The structure reveals the fine molecular details of IgE 2F10 binding its 750 Å^2^ conformational epitope on Der p 2.

**Summary:**

This review provides an overview of this major milestone in allergy, the first atomic resolution structure of an authentic human IgE epitope. The molecular insights that IgE epitopes provide will allow for structure-based design approaches to the development of novel diagnostics, antibody therapeutics, and immunotherapies.

## Introduction

At the center of the pathogenesis of allergic diseases is the IgE antibody molecule. In sensitized individuals, re-exposure to the allergen results in IgE engagement, causing Fcε receptor cross-linking and activation of mast cells and basophils. This triggers the release of mediators into the local tissue, culminating in the broad array of immediate symptoms associated with allergy and contributes to chronic allergic inflammation. To date, studies of the human IgE molecule, and its targeted epitopes on allergens, have been very limited. Most of our knowledge of this process has come from studies using allergic patient sera and surrogate monoclonal antibodies. Because serum contains a mixture of many antibodies, having many specificities, directed toward many different epitopes, and having heterogenous affinities, the ability to study the molecular interaction of human IgE with its target allergen by this method is limited. The ideal way to study this process is to use naturally occurring human IgE monoclonal antibodies (mAbs), that is, those antibodies which occur naturally as part of the human immunoglobulin repertoire, isolated from allergic patients. Unfortunately, until very recently, no such antibodies had ever been made. The field of molecular allergy, however, has worked for many decades on defining the allergen; making, and defining hundreds of the most common disease associated allergens. This is an invaluable asset, now allowing for the rapid identification and characterization of allergen-specific human IgE molecules.

To have the ability to use naturally occurring human IgE mAbs (hIgE mAb) to study the epitopes at the center of allergy pathogenesis has been desired for many decades. There are three principal reasons why these antibodies have not been developed. The first reason is that techniques for efficiently making full-length (including the naturally occurring Fc) human mAb were not in place until recently. The second reason is that the frequency of IgE producing memory B cells in peripheral blood is exceedingly low [1], even in allergic individuals. Techniques to make full-length naturally occurring hIgE mAb rely on the availability of peripheral blood B cells which encode the IgE antibody of interest. The third reason is the difficulty in identifying and growing IgE-producing B cells in primary culture. There is strict regulation of IgE B cell receptor expression and soluble antibody secretion. This results in great difficulty identifying IgE B cells within peripheral blood mononuclear cell (PBMC) samples by fluorescent labeling the B cell receptor [2, 3]. Primary cultures containing one clone of B cell expressing IgE produce levels below detection with even the most sensitive ELISA. For this reason, studies of IgE expressing B cells in culture have only involved artificially class-switched polyclonal cultures using the cytokine IL4 [4, 5]. Since IL4 class-switches B cells in a nonpredictive way, allergen-specific human antibodies are not made.

Advances in hybridoma technologies have allowed for the first time the identification, amplification, and ultimately the generation of stable cell lines, human hybridomas, from the very rare population of IgE expressing B cells present in peripheral blood. With the ability to generate full-length naturally occurring hIgE mAb, we can now proceed with studies of the most basic, and critical, molecular interactions that make up the heart of allergy pathogenesis and disease. In addition, the antibody and allergen tools/reagents, and the many insights that they provide, will allow for the design and development of novel diagnostics, therapeutics, and immunotherapies.

## Allergen-Specific Polyclonal Serum

Despite IgE causing an abundance of human disease, it was not until 1967 that the “reagin” molecule was discovered [6, 7]. This is due to its very low serum concentration relative to other antibody isotypes—over one hundred thousand-fold less than IgG in healthy individuals. Only one IgE secreting cell line (U266), or its derivatives (SKO-007), was available to study. U266 is a lymphoblast plasmacytoma established from the peripheral blood of a 53-year-old man with IgE-secreting myeloma (ND) in 1968, described in the original paper [7, 8]. The IgE molecule produced by these cells has been of integral importance, used in thousands of studies as a reagent or for the generation of reagents. However, neither the target antigen for ND nor for another commonly used IgE myeloma protein (PS) has ever been identified, highlighting the need to use IgE of known specificity from serum [9, 10]. Nearly all our understanding of the human IgE antibody has come from the very low concentration of polyclonal antibody found in allergic patients’ serum. Serum contains a mixture of antibodies, which have many protein specificities, target untold numbers of epitopes, and have different affinities. This results in the inability to accurately study the molecular interaction of IgE with its target allergen(s). Furthermore, polyclonal populations of IgE antibodies in serum are mixtures of different concentrations of allergen-specific and cross-reactive antibodies. Allergen-specific IgE measurements in serum cannot distinguish between these individual populations, those that are allergen cross-reactive from those that are allergen-specific but having low affinity.

## Allergen-Specific B Cells

When common staining methods are employed to measure IgE B cells using flow cytometry, nonspecific surface-bound IgE may obscure the analysis because non-IgE-expressing cells stain positively due to IgE bound to CD23, present on all B cells [1, 11]. Studies using circulating B cells that have been stripped of their surface proteins by proteolysis, followed by labeling of internal IgE using fixation/permeabilization, have generated more reliable frequencies [12]. In fact, there are no studies that accurately provide information about the frequencies of allergen specific IgE encoding clones within the peripheral blood of an allergic patient, nor can they provide information about the genetic lineage of this IgE B cell population. There remains much uncertainty regarding the durability and location of the IgE memory B cell response. Much of our understanding comes from murine studies, and it is difficult to determine how much of this can be applied to humans [13]. Deep sequencing data, however, suggests IgE memory B cells are present in circulation, as antibody sequences can be obtained and arise from common germlines in an unbiased manner [14].

Since the technologies to make naturally occurring human IgE have not existed previously, investigators have used alternate strategies to gain insights into to human allergen specific IgE antibody response. Recombinant chimeric human mAb have been generated by expressing allergen-specific murine variable gene sequences with human IgE constant region [15–17]. Christensen et al. performed some of the most informative studies using humanized dust mite-specific murine mAb, showing how antibody concentration, affinity, and the antigenic site influence basophil degranulation [18]. This group used these synthetic mAb as tools to indirectly assess the complexity of dust-mite sensitized human sera [19]. Chimeric molecules are useful tools to study how cell bound IgE-allergen complexes mediate degranulation, but do not provide much information about the human antibody response to the allergen given their murine origin of the variable domains.

Naturally occurring human allergen-specific IgG mAb have been isolated from individuals receiving immunotherapy [20–22]. These mAb may help to clarify the mechanism by which immunotherapy leads to improvement in clinical symptoms, but they are unlikely to provide insights into the IgE repertoire and its interactions with the allergen that leads to allergic disease. Some understanding of the human allergen specific antibody responses has come through use of phage display techniques. Monoclonal antibodies generated by phage display have the human heavy chain variable sequence obtained using IgE specific primer sets, but are not considered naturally occurring IgE molecules (they do not have the natural light chain pairing). Despite having a heavy chain variable sequence that is found to bind a specific allergen, they are unlikely to possess the native light chain (which may participate in binding) and do not provide any information about the IgE constant region [23, 24]. All the allergen-specific IgE antibody work performed to date is nicely described in a recent review article by one of the leaders in the field [25]. The ideal approach to study the naturally occurring IgE B cell response and the antibody it encodes is to be able to grow and examine the memory B cell itself—this can now be done by making human hybridomas.

## Generation of Naturally Occurring hIgE mAb

To date, two techniques have been recently published that successfully capture the naturally occurring IgE antibody, using human hybridoma and single cell RNA sequencing technologies [26•, 27•]. Croote et al. describes the use of modern sequencing technologies to identify and characterize rare IgE encoding peanut allergen specific B cell clones within the peripheral blood of peanut allergic subjects. Not only did this technology allow for the generation of naturally occurring peanut-specific IgE mAb, but it also showed the existence of ultra-rare IgE encoding memory B cells and plasmablasts in the peripheral blood. IgE sequences then were expressed as recombinant human IgG antibodies and used for binding analyses against peanut allergen. The generation of human hybridomas has also been used to study human antibody responses and has been employed for the development of antigen-specific IgE monoclonal antibodies. This technology, though exceedingly labor intensive, preserves the entire antibody molecule, including the Fc and its native glycosylation. Antibodies made using hybridoma technology are expressed from the subject’s B cell nucleus without any recombinant manipulation. If, however, recombinant expression is desired the sequence can be obtained using standard RT-PCR techniques [28].

**Fig. 1:**
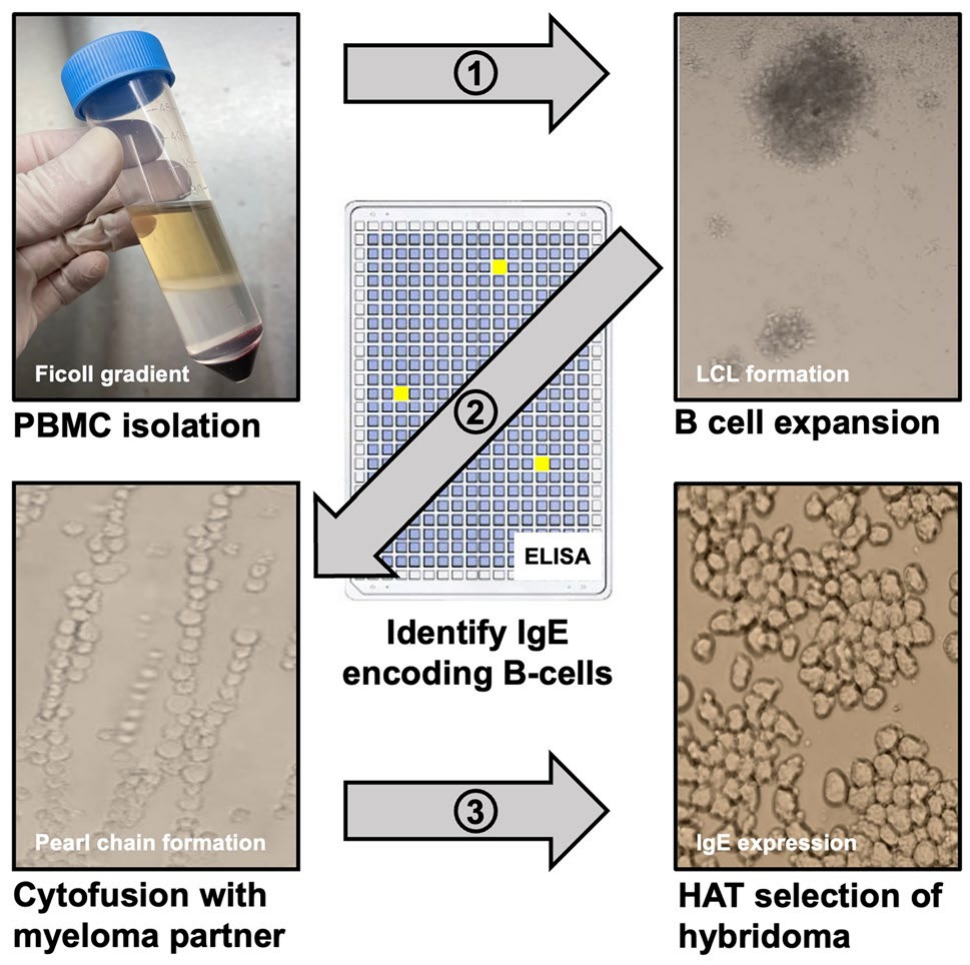
IgE secreting hybridoma generation overview. PBMCs are isolated from selected subject’s blood using a Ficoll density gradient method. In step 1, cells are grown in 96-well tissue culture plates for 7 days to expand all B cells, leading to the development of lymphoblastoid cell lines (LCLs). Cultures containing IgE are identified by ELISA and cells within wells containing IgE are fused with a myeloma partner by electrical cytofusion reactions in step 2. Human hybridomas are selected for in HAT medium in step 3 and biologically cloned using flow cytometric single cell sorting. Finally, IgE secreting hybridomas are grown in serum free culture medium and IgE monoclonal antibody purified by omalizumab chromatography

Historically, it has been easy to make murine mAb using hybridoma technologies, but extremely difficult to make hybridomas to selected targets using human peripheral blood B cells. Improvements in the efficiency and versatility of human hybridoma generation have enabled us to generate hundreds of naturally occurring human IgG mAb using hybridoma technology [29–32]. However, it was advances made recently which have resulted in our ability to move this technology to successfully generate for the very first time human hybridomas secreting IgE mAb. For detailed methods of how IgE secreting hybridomas are generated from PBMC, see Wurth et al. [26•] (Fig. 1).

Several specific advancements allowed IgE secreting hybridomas to be generated. First, and most importantly, IgE encoding B cells were expanded in primary culture in only 7 days. IgE encoding memory B cells do not expand sufficiently using Epstein–Barr virus (EBV) transformation [33, 34]. This has only been achieved using the NIH3T3 fibroblast line that was genetically engineered to constitutively express cell-surface human CD154 (CD40 ligand), secreted human B cell activating factor (BAFF), and human IL-21 (created by Dr. Deepta Bhattacharya; University of Arizona). Allergic patient PBMCs are grown in the presence of gamma-irradiated NIH3T3 fibroblasts and their B cell receptors are globally cross-linked and activated using a mixture of murine anti-human light chain mAb. The kinetics and efficiency of human B cell activation and expansion is improved with TLR9 stimulation by CpG oligodeoxynucleotides and the inhibition of apoptosis with the addition of a pharmacologic inhibitor of the serine/threonine-protein kinase Chk2, which is required for checkpoint-mediated cell cycle arrest [35]. The second essential tool needed for production of IgE secreting hybridomas is to have the ability to accurately detect very rare populations of B cells that secrete low ng/mL concentrations of IgE in primary culture. Identification of wells containing IgE is achieved by using a mAb sandwich ELISA, where both capture and detection mAb are human IgE-specific. Adequate sensitivity is achieved by using a secondary antibody conjugated to horseradish peroxidase (HRP) and a fluorescent substrate.

## Identification of Rare Allergen-Specific Human IgE Encoding Cells

We have generated > 600 naturally occurring human IgE secreting hybridomas. Using patient serum IgE profiles and skin prick testing results, obtained by their allergist, we can identify subjects which not only have high titers of circulating IgE, but also circulating IgE encoding memory B cells. Hamilton et al. described the relationship between total serum IgE and allergen-specific IgE concentrations for several important allergens using 18,950 paired readings [36]. This information provides the best clues to what the expected proportion of the memory B cell response is directed toward a particular allergen. In other words, for example, how often do you find subjects with cat allergy for which more than 4% of their total IgE is directed toward cat allergens. Interestingly, 27% of individuals, not accounting for age, had approximately 1/20th of their total IgE directed toward cat. This data suggests that if subjects are selected based on their serum IgE profiles, allergen specific B cell clones can be enriched, making them more detectable and available for study. The hIgE mAb can be generated without screening B cell cultures for allergen specificity. Having blood cells from subjects with the allergic disease we wish to study, allows for the enrichment of allergen specific IgE memory B cells in their peripheral blood, and a high probability of capturing the specificity driving their disease. Thus, by selecting the right human subjects, we can identify the desired allergen specificities without needing to screen B cell cultures for allergen specificity.

## Molecular Parameters of IgE-Allergen Interactions That Dictate Downstream Effects

A very important concept at the heart of allergic disease is the antigenic site (antigenic region) where IgE binds. For cross-linking of Fcε receptors to occur with a native monomeric allergen protein, two different IgE antibodies must bind simultaneously to two different antigenic sites on the allergen [37–39]. In contrast, for some multimeric allergen proteins, the same antibody might cross-link Fcε receptors when it binds to the same site on each of the allergen protomers forming the multimeric assembly [40, 41]. The cross-linked IgEs physically pull together two Fcε receptors, allowing for signal transduction to take place. This implies that the relationship of antigenic sites to one another plays an important role in the potency of receptor cross-linking, as sites that are in proximity could bring Fcε receptors closer together [37, 38]. Antigenic sites can be easily defined by antibody competition assays [42]. Naturally occurring hIgE mAb can be used to define antigenic sites on allergen proteins and can be tested in combinations to determine which antigenic sites might result in the greatest receptor cross-linking. Spatial constraints determined by the allergen three-dimensional structure, such as the distance between different IgE antibody binding epitopes on the allergen, determine productive downstream signaling that leads to cellular degranulation [43, 44]. Molecular constraints may help explain why many of the most important allergen proteins are small, < 50 kDa.

An additional consideration that IgE antibody molecules provide, which influence the strength of receptor cross-linking and the downstream effects of cell activation, is their affinity of binding [45]. The kinetics (k_on_, k_off_, K_D_) of IgE-allergen protein interactions can only be measured using monoclonal preparations of antibody. Using murine IgG antibodies expressed as chimeric human IgE constructs, to act as a surrogate of human IgE antibodies, it was found that higher affinities possess more functional potency [18]. Taken together, it seems likely that the most potent allergic reaction inducing IgE molecules bind epitopes located within close neighboring antigenic sites and have very high affinity for their allergen target protein [37–45]. Considering the complexity of the human serum IgE antibody profile, such molecular parameters must all be at play in determining the overall receptor cluster size resulting in the signals that influence the cells’ effector responses to allergen stimulation [38].

## Advantage of Human IgE Monoclonal Antibodies for Epitope Mapping

Allergen-IgE interactions are key for the development of allergic disease. The interactions occur either at the level of facilitated antigen presentation by dendritic cells, or at the level of induction of an allergic response, by cross-linking IgE bound to basophils and mast cells, which leads to induction of mediator release. Therefore, mapping of IgE antibody binding sites or epitopes on allergens provides insight into the IgE antibody repertoire and the allergic response. Epitope mapping allows the specificity and cross-reactivity of homologous allergens to be elucidated, which has its implications for diagnosis and immunotherapy. Finally, by locating IgE antibody binding epitopes, hypoallergens with reduced capacity to bind IgE can be designed, with the goal to reduce side effects resulting from increasing allergen doses during immunotherapy. The recent availability of human IgE monoclonal antibodies has opened new possibilities for precise epitope mapping using X-ray crystallography.

To provide a historical perspective, several approaches to map IgE epitopes are reviewed, based on literature published recently (Table 1). These are experimental approaches that do not include in silico predictions [46]. Original IgE mapping studies in the 1990s tested overlapping peptides covering the full sequence of the allergen, enzyme digests or recombinant allergen fragments for their capacity to bind IgE [47, 48]. This approach has evolved over time with the development of peptide microarrays [49–51, 52•, 53] and bead-based assays [54–56], which also measure antibody binding to peptides bound to different solid phases (e.g., glass slides or polystyrene beads instead of the original nitrocellulose or sepharose). These approaches have allowed high throughput analysis of large numbers of peptides. Recently, a programmable phage display library, AllerScan, composed of peptides from all the protein sequences present in the Allergome database (~ 2000) was created and used to characterize IgE and IgG antibody reactivities in a cohort of food allergic individuals [57•, 58]. An extensive overlap between peanut oral immunotherapy-induced IgE and IgG4 specificities was observed, as well as similar antibody footprints, suggesting related clonal lineages or convergent evolution of IgE and IgG B cells [58].

**Table 1:** Analysis of allergen-IgE antibody interactions

Approach	Antibody clonality	What is identified	Example
Test IgE binding to synthetic peptides or small fragments on cellulose or microarrays or bead-based assays	Polyclonal or monoclonal	Linear epitopes	47–67
Site-directed mutagenesis in peptides	Polyclonal or monoclonal	Specific antibody binding residues in linear epitopes	69
Phage display technology		Mimotopes	73,74
Testing antibody binding to chimeras or hybrid molecules that result from grafting allergen surface areas onto non-allergenic homologous proteins	Polyclonal	Allergen surface patches containing conformational (or linear) epitopes	75,76
Nuclear magnetic resonance (hydrogen/deuterium exchange memory; methyl-labeled allergen in complex with mAb)	Monoclonal	Identification of area containing a conformational epitope	77,78
X-ray crystallography of allergen with an IgG mAb construct as surrogate of human IgE, followed by site-directed mutagenesis analysis	Monoclonal	Identification of residues involved in IgG and IgE antibody binding	70
X-ray crystallography of allergen in complex with a human IgE mAb construct	Monoclonal	Detailed structure of the allergen-IgE interaction	80

Similarly, a global microarray platform representing all possible linear overlapping 16-mer epitopes (> 17,000) of 731 allergens defined by the World Health Organization and the International Union of Immunological Societies (WHO/IUIS) Allergen Nomenclature Sub-Committee was generated [59•]. The platform was used to identify development of recognition of linear epitopes by IgG, IgG4, and IgE in sera/plasma during 3 years of allergen immunotherapy against birch and grass pollen allergy [59•].

Some of the peptides identified in these studies have been reported to be useful to assess the epitope-recognition profiles of antibody repertoires for different isotypes [60, 61], for diagnosis [52•, 55, 62•, 63] or to stratify patients regarding sensitivity to challenge and outcome of oral immunotherapy [51, 64•, 65]. Machine learning algorithms combining epitope and antigen-specific IgE levels in the first 2 to 3 years of life improved the accuracy of predicting peanut allergy at age 4 + years [66]. In addition, linear epitopes recognized by different isotypes can be compared. Similar binding patterns were found for IgE and IgG4 among lentil allergic patients, as had been previously found for milk, peanut, and egg allergy [67]. The intensity of binding by both antibody isotypes was higher in reactive than outgrown lentil patients for each positive epitope. These results show that during acquisition of natural tolerance the IgE and IgG4 antibody levels decrease, and there is an important association between both antibody isotypes.

Limitations of these approaches are the lack of posttranslational modifications in the peptides and the focus on only linear epitopes by design. Linear epitopes are consecutive amino acids in the allergen sequence recognized by IgE.

While linear epitopes are common in food allergens due to their processing in the digestive tract and/or before ingestion (e.g., thermal treatment), conformational or discontinuous epitopes also exist in foods [50, 63], and are the most common epitopes in inhalant allergens. Conformational epitopes involve amino acids that are non-contiguous in the amino acid sequence but are close in the space due to the protein folding. Analysis of conformational epitopes is more demanding and requires approaches that include as a pre-requisite the three-dimensional structure of the allergen. For years, due to the lack of human IgE monoclonal antibodies, indirect ways to define conformational epitopes have been used:

1. A reduction of IgE antibody binding to modified allergen molecules in dot blots or enzyme-linked immunosorbent assays (ELISA) [68, 69].
2. Relative epitope mapping using an antibody of known epitope specificity for capture of the allergen and the IgE for detection [70]. Based on a similar principle, “epitope masking” can be used for epitope mapping, by creating a panel of mutant antigens in which single surface-exposed residues are mutated to cysteine. The cysteines are chemically tethered to a solid phase, thereby masking a surface patch centered on each cysteine residue and blocking the binding of antibodies to this region of the surface [71].
3. Raising rabbit antisera against surface-exposed allergen derived peptides. Blocking of binding of allergic subjects’ IgE to the complete folded allergen by the antisera indicates the presence of a conformational epitope [72].
4. Mimotopes or peptides that mimic IgE epitopes have been identified by phage display technology [73, 74]. Peptides recognizing human IgE are selected from phage display libraries and are located (using a computational algorithm and the allergen structure) on allergen surface patches that mimic conformational epitopes, but are not the same as the real epitope.
5. Creating hybrid molecules or chimeras, by grafting an area containing conformational or linear epitopes into a homologous nonallergenic molecule. Conformational IgE epitopes have been localized on surface patches of the allergen by comparing IgE binding to the chimeras [75, 76•].
6. Using NMR-based hydrogen/deuterium exchange to identify areas containing conformational epitopes. Three conformational epitopes for murine mAb were identified in the lipid transfer protein Art v 3. Variants with mutated epitopes showed reduced binding of IgE from serum of allergic patients, which indicated the importance of the targeted residues for IgE recognition [77].
7. X-ray crystallography of an allergen with an IgG mAb construct as surrogate of human IgE, followed by site-directed mutagenesis analysis [70].

With the advent of hIgE mAb, direct epitope mapping can be performed. Four hIgE mAb were isolated that are specific for the mite allergen Der p 2. Immunoassays showed that three of them (1B8, 5D10, and 2G1) overlapped with a murine IgG mAb (α-DpX), whereas a fourth (2F10) bound at a more distal position in the molecule. An NMR approach using methyl labeled Der p 2 identified two areas containing the epitopes for the four hIgE mAb [78]. Additional technologies that also consider the three-dimensional structure of proteins for epitope mapping are cryo-electron microscopy (cryo-EM) and chemical protection assays combined with mass spectrometry (MS) [79]. Human IgE monoclonal antibodies allow a precise and detailed analysis of the allergen-IgE interaction by the determination of the X-ray crystal structure of an allergen with an antibody construct, which provides the most accurate information of the interaction [80••].

## X-Ray Crystal Structure of hIgE mAb in Complex with an Allergen

**Fig. 2:**
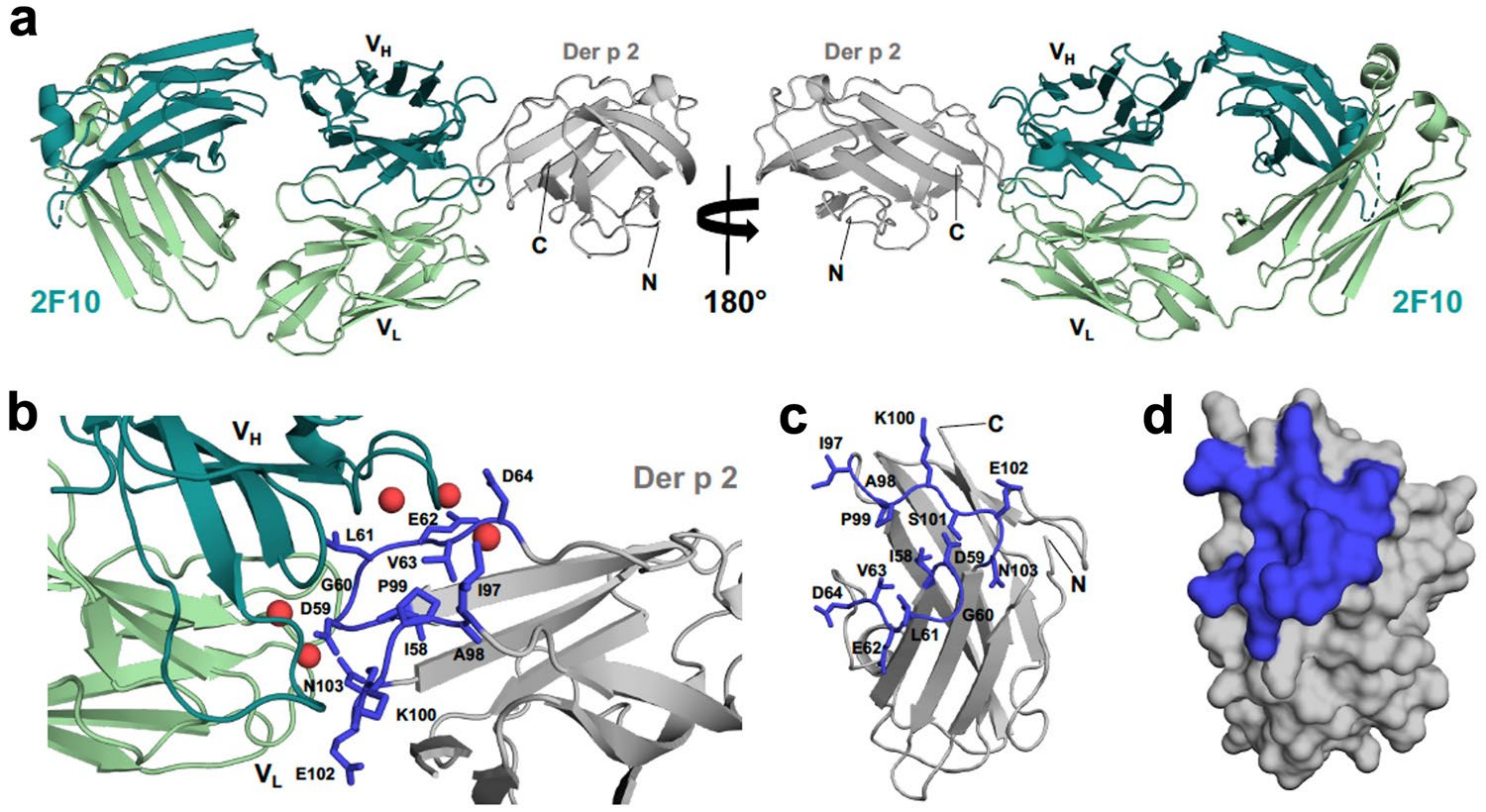
Complex between Der p 2.0103 and IgE 2F10 Fab. **a** Der p 2 and 2F10 Fab are shown in ribbon representation. Heavy chain of the antibody is shown in teal, while light chain is shown in pale green. **b** Interface between 2F10 and Der p 2. Residues forming the epitope are marked in blue and their side chains are shown in stick representation. 2F10 binding epitope (in blue) mapped on the structure of Der p 2 shown in ribbon **c**, and space filling **d**, representations. Water molecules are represented by red spheres. N- and C-terminal ends of Der p 2 are labeled in **a** and **c**

X-ray crystallography provides precise structural detail of the interactions between allergens and antibodies. Mapping of an epitope requires crystallization of the allergen with a fragment of an antibody (usually Fab), as the whole antibody is conformationally flexible and therefore difficult to crystallize. Currently, only one X-ray crystal structure of a complex of an allergen (Der p 2) with a human IgE mAb construct that has the correct pairing of the heavy and light chains has been reported (PDB code: 7MLH) [80••]. Previously, two structures of human IgE Fabs in complexes with Bos d 5 (PDB code: 2R56) or Phl p 2 (PDB code: 2VXQ) were published [40, 81]. In both cases, the antibody constructs were derived from phage display combinatorial libraries. Therefore, the heavy-light chain pairing was not necessarily the same as that present in vivo. Recently, two other PDB deposits reveal structures of complexes between murine IgE Fab and Hev b 8 (PDB codes: 7SBD and 7SBG) [82]. From these five structures of IgE in complex with allergen, we will focus on Der p 2 in complex with Fab from the hIgE mAb 2F10 (2F10 Fab) (Fig. 2a–d). The interface between Der p 2 and 2F10 Fab has an area of approximately 750 Å^2^, which is lower than average for currently known allergen-antibody complexes [80••]. Like other allergen-antibody complexes, 2F10 heavy chain contributes most of the surface area to the interaction with the allergen. The interactions (number of residues and hydrogen bonds) between epitope and paratope observed for the Der p 2–2F10 Fab complex are quite similar to those observed for IgG antibody/antigen or antibody/allergen complexes [80••]. This is not surprising because the sequence of IgE 2F10 is only moderately mutated compared with the germline sequence [80••]. Der p 2 residues 58–64 and 97–103 form a conformational epitope that is recognized by IgE 2F10 (Fig. 2b–d). There are five water molecules trapped between the allergen and antibody which bridge interactions between protein molecules (Fig. 2b). Presence of these water molecules is not unusual and often improves the fit between antigen and antibody surfaces [70, 83, 84]. The IgE 2F10 recognizes Der p 2 homologues from other house dust mite species (Der f 2, Eur m 2), but does not bind Group 2 allergens from storage mites (Gly d 2 and Lep d 2). This is due to low amino acid sequence identity between Der p 2 and the homologous allergens from storage mites, especially at the corresponding areas of the hIgE mAb 2F10 epitope [80••]. Mutations of the 2F10 binding epitope revealed that residues Asp59, Leu61, and Lys100 (Fig. 2b, c) are especially important for IgE antibody binding, which was also confirmed by in vivo studies.

**Fig. 3:**
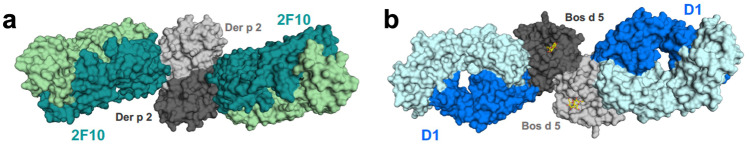
**a** 2F10 Fab binding to a putative Der p 2.0103 dimer. **b** D1 IgE Fab in complex with Bos d 5 dimer. Non-physiological Bos d 5 ligand (dodecyl-β-D-maltoside) is shown in stick representation

The crystal structure of the Der p 2–2F10 Fab complex also shows that Der p 2 may form dimers (Fig. 3a). The dimeric Der p 2 assembly that can be identified in the structure of the complex is almost the same as the dimer present in the crystal structure of uncomplexed Der p 2 (PDB code: 1KTJ) [85]. This is consistent with gel filtration chromatography results that clearly indicate that both Der p 2 monomers (primarily) and dimers are present in solution (especially in concentrated allergen solutions) [80••]. Such allergen dimerization was observed in the complex of Bos d 5 with IgE Fab (Fig. 3b) and in the complex of Bla g 2 with IgG Fab [40, 86]. While the allergen oligomerization process may have a profound impact on cross-linking the high affinity IgE receptors (FcεRI) and triggering allergic reaction, it is not clear whether such a process takes place in vivo for Der p 2.

The epitope-containing area recognized by hIgE mAb 2F10 was identified by NMR, in conjunction with the binding areas for three other hIgE mAb (1B8, 2G1, and 5D10) [78]. In addition, several murine IgG mAb (7A1, 1D8, and αDpX) bind Der p 2 and their epitopes are known [70, 78]. Therefore, currently Der p 2 is the best studied allergen regarding details of its interactions with antibodies, especially IgE. In future, many more structures of allergen-IgE complexes will be deposited to the PDB, which will provide explanation of the molecular basis of events leading to allergic reactions and enable production of hypoallergens for immunotherapy.

## Research and Diagnostic Applications of hIgE mAb

The hIgE mAb technology platform has made available panels of antibodies to inhaled and food allergens which provide tools for research and diagnostic applications. Currently, the hIgE mAb include a diverse array of subject derived antibodies to:

- Inhalants—Der p 1, Der p 2, Fel d 1, Can f 1, Can f 6
- Foods—Ara h 1, Ara h 2, Ara h 3, Ara h 6, Gal d 1, Gal d 2, alpha-gal, Jug r 1, Ana o 3

This is a remarkable collection of reagents. Typically, serum IgE levels are considered high at ImmunoCAP values of class 6 or ~ 100 kUA/L specific IgE antibody. The concentrations of hIgE mAb are several orders of magnitude greater and range from 10,000 to > 500,000 kUA/L IgE. These IgE antibody levels exceed those of even the most highly allergic patients. Several hIgE mAb (to Der p 2, Fel d 1, Ara h 2, Ara h 6) recognize non-overlapping epitopes on the allergen which is useful for mechanistic studies. Structural studies of the hIgE mAb/allergen complexes for all these allergens will enable the IgE epitope(s) to be localized. The recent studies with hIgE mAb 2F10 and Der p 2 can be extended to other allergens to define the repertoire of IgE epitopes on these molecules and to answer a fundamental question that has eluded allergists for many years “What do IgE antibodies recognize and what constitutes an ‘allergenic’ epitope?” This is especially relevant for food allergens where there is increasing debate about the role of linear or conformational epitopes in the etiology of food allergy and in the development of tolerance to food allergens. Determining the structure of hIgE mAb complexes with peanut, milk and egg allergens will provide definitive data on the epitopes involved.

The hIgE mAb have been validated for allergen specificity by ELISA, by immunoblotting using purified allergens and for antibody reactivity by ELISA, ImmunoCAP, and total IgE assays (unpublished data). The biologic activity of hIgE mAb to Der p 2 has been demonstrated in a mouse model of anaphylaxis [80••]. The biologic activity of hIgE mAb to other allergens (Fel d 1, Der p 2, Ara h 2, Ara h 6) is being investigated by analyzing mediator release assay using rat basophilic leukemia cells transformed with the human FcɛRI for passive mAb sensitization (in collaboration with Dr. Lorenz Aglas, University of Salzburg, Austria). Preliminary data have identified pairs of hIgE mAb to Fel d 1 and Der p 2 that will induce allergen specific mediator release in this assay and experiments with Ara h 2 and Ara h 6 are in progress.

The momentum in in vitro allergy diagnostics is firmly in the realm of component resolved or allergen molecules as improved alternatives to allergenic extracts. Measuring IgE antibodies to Ara h 2 has greater diagnostic efficacy than using peanut extract [87]. Molecular IgE testing is also being used to differentiate allergens of clinical significance from those that reflect spurious cross-reactivity. For example, patients who are allergic to birch pollen may have cross-reactive IgE antibodies to peanut allergens (Ara h 5 and Ara h 8) and give positive skin tests to peanut extract but are at very low risk of having allergic reactions on eating peanuts. The clinical significance of using allergen molecules in allergy diagnostics is thoroughly reviewed and updated in the latest edition of the Molecular Allergology User’s Guide 2.0 (MAUG 2) which has just been released (July 2022) [88••]. Prepared under the auspices of the European Academy of Allergy and Clinical Immunology (EAACI), MAUG 2 includes expert reviews of the use of allergen molecules in clinical practice and is highly recommended. The manufacture of high-quality purified allergens is a key element in molecular testing and requires a large repertoire (120–170 purified allergens depending on the device or platform technology being used). A significant challenge for developing new allergy diagnostic systems is the limited availability of sera from allergic patients for validation purposes. Each allergen requires sera with a range of specific IgE antibody levels from 50 + well-defined allergic patients. Obtaining these sera is time consuming and expensive. It is especially difficult for allergens that primarily affect pediatric populations, such as milk and egg. The hIgE mAb can be used for quality control and validation of the purified allergens used in molecular diagnostics. They are definitive probes for allergen identification and enable manufacturers to confirm the allergenic integrity of the purified natural or recombinant allergens used in molecular testing. The hIgE mAb provide useful alternatives to human sera for developing diagnostic tests and can potentially be used as calibrators and standards for in vitro testing. Ultimately, it should be possible to develop a national or international standard(s) for IgE using one or more of the hIgE mAb. Finally, the use of hIgE mAb to different allergen epitopes could serve as controls for basophil activation tests, which are increasingly being considered as alternatives to food challenges for food allergy diagnosis [89].

## Conclusions

Allergic diseases are a growing global health concern, with food allergy leading the way. At the helm, IgE antibodies bound to their high affinity receptor on mast cells and basophils that initiate cell activation and mediator release upon allergen recognition. Most of our knowledge of these allergen-specific IgE antibody molecules has come from studies using IgE found in patient serum. Due to the polyclonal nature and exceedingly low concentration of IgE in human serum, the ability to study the fine molecular details of the antibody-allergen interaction is limited. Alternate antibody approaches have been employed, but until recently no naturally occurring allergen specific human IgE monoclonal antibodies have existed. Thanks to advances in human hybridoma technology, IgE mAb can now be generated from patients with allergy. The specificities of the IgE mAb generated using this technology reflect the IgE sensitizations and allergic diseases of the patient from which they were derived. These unique antibody tools can be used to obtain highly detailed insights into their specific interactions with their disease-causing allergen. IgE mAb secreted from human hybridomas are full-length, including the Fc, as they are expressed from the patient’s B cell’s nucleus. IgE mAb variable gene sequences can be amplified and expressed as isotype switch variant immunoglobulins or as molecular Fabs for use in structural studies. Using the IgE variable gene sequences of Der p 2 specific antibody 2F10, the first authentic IgE epitope was determined using X ray crystallography. IgE mAb 2F10 was found to bind a conformational epitope of approximately 750 Å^2^. Having an atomic resolution structure of the interface between antibody and allergen allows for the creation of mutant allergens that are no longer recognized by the IgE mAb, a technique often referred to as structure-based antigen design. Mutation of the 2F10 epitope on Der p 2 established that residues Asp59, Leu61, and Lys100 are especially important for IgE antibody binding and function, also confirmed by in vivo studies. The ability to now study human IgE mAb hereby marks a major milestone in the allergy field, where authentic epitopes can be determined, and structure-based design of hypoallergens can be pursued. These human IgE mAb will contribute to analyze the human IgE antibody repertoire and associated epitopes, and lead to the development of new molecules for diagnosis and therapy of allergic diseases.
